# Artificial intelligence for precision nephrology: current evidence to clinical translation and future prospects

**DOI:** 10.3389/fneph.2026.1950125

**Published:** 2026-10-30

**Authors:** Moses Ishaku Madami, Chigozirim Samuel Amaoge, Ayu Wulandari, Xiaoshuang Zhou

**Affiliations:** 1 The Department of Nephrology, Shanxi Provincial People’s Hospital, Shanxi Medical University, Taiyuan, China; 2 School of International Education, Shanxi Medical University, Jinzhong, Shanxi, China; 3 Shanxi Provincial Key Laboratory of Kidney Disease, Taiyuan, China; 4 Medicinal Basic Research Innovation Center of Chronic Kidney Disease, Ministry of Education, Shanxi Medical University, Taiyuan, China

**Keywords:** artificial intelligence, health equity, kidney disease, machine learning, nephropathology, precision medicine

## Abstract

**Background and objectives:**

Artificial intelligence (AI) is increasingly being applied across nephrology, with potential applications spanning disease prediction, diagnosis, treatment optimisation, and clinical decision support. However, reported model performance varies substantially across clinical domains, and high predictive performance does not necessarily demonstrate clinical utility or readiness for deployment. This narrative review synthesizes current evidence on the applications of AI in nephrology and critically evaluates the methodological maturity, validation, interpretability, fairness, and translational readiness of AI systems across major nephrology domains.

**Methods:**

A structured literature search of PubMed was conducted using terms related to AI and nephrology, with the primary eligibility period restricted to studies published from 2020 to 2025. The search strategy incorporated artificial intelligence, machine learning, deep learning, neural networks, natural language processing, large language models, generative AI, computer vision, reinforcement learning, federated learning, and related terms, together with nephrology and kidney-disease terms. Title/abstract and full-text screening were performed independently by the first and second authors, with disagreements resolved by the third author. Two foundational pre-2020 studies identified through citation chaining were retained because of their importance to the subsequent development of AI applications in nephrology. Evidence was synthesized qualitatively according to study design, validation, predictive performance, interpretability, fairness, prospective evaluation, and translational readiness.

**Results:**

AI applications were identified across acute kidney injury, chronic kidney disease, nephropathology, kidney transplantation, haemodialysis, peritoneal dialysis, large language models, reinforcement learning, and federated learning. Across these domains, many models demonstrated promising discrimination, but evidence maturity varied considerably. External validation was present for some applications, whereas prospective evaluation and evidence of clinical utility remained limited. The literature also highlighted challenges related to calibration, generalizability, interpretability, algorithmic fairness, data governance, and integration into clinical workflows. Large language models and federated learning represent emerging areas, but nephrology-specific evidence remains comparatively limited.

**Conclusions:**

AI has substantial potential to support precision nephrology, but technical performance should not be equated with clinical effectiveness or deployment readiness. Future research should prioritize external and prospective validation, assessment of clinical utility and patient outcomes, transparent reporting, fairness, regulatory oversight, and effective human–AI integration.

## Graphical Abstract

**Figure:**
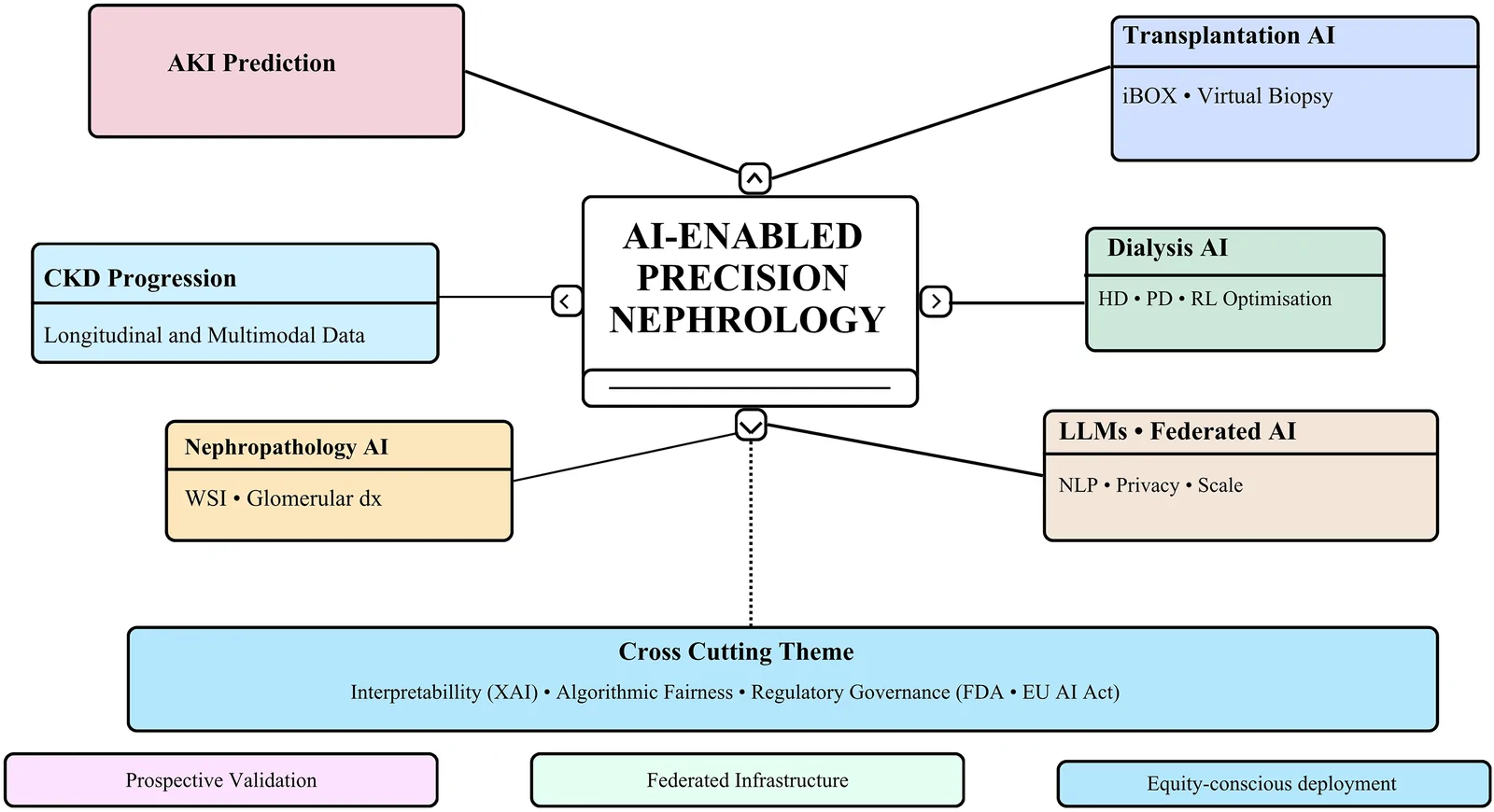
The organizing structure of this review is depicted in the graphical abstract. AI applications converge towards a central precision nephrology sector.

## 1 Introduction

Nephrology disease conditions and a range of rare nephropathies jointly represent a biologically heterogeneous disease spectrum that conventional diagnostic tools and even recent AI methods have failed to fully capture. CKD and AKI, whether occurring concurrently or independently, impose substantial burdens on patients, health systems, and clinicians, particularly in intensive care settings (1, 2).

Precision nephrology integrates histopathological, clinical, imaging, and longitudinal patient data to provide customized risk prediction, including therapeutic and adaptive management and disease phenotyping. Beyond the technical performance, precision nephrology also needs to be applied fairly across a range of demographics to guarantee that AI-driven advancements improve outcomes without worsening already-existing healthcare disparities.

Over the past five years, both machine learning (ML) and deep learning (DL) architectures have demonstrated strong performance in identifying AKI up to 48 hours prior to biochemical manifestation (3, 4), predicting the progression of chronic kidney disease (CKD) through multimodal clinical integration (5, 6), classifying kidney biopsy pathology with accuracy comparable to expert pathologists (7, 8), stratifying allograft risk at molecular precision (9), and providing clinical documentation support and reasoning that approaches nephrology expert-level performance (10–14). AI’s widespread clinical applications in nephrology are still inadequate, although these advancements collectively show that it has the potential to revolutionize nephrology’s diagnostic, prognostic, and therapeutic decision-making. A recurrent disturbing pattern pervades the current literature, models that demonstrate exceptional internal validation often fail under external testing, and the tools trained on data-rich, predominantly White male cohorts fail in diverse populations. Clinicians also distrust black box outputs they cannot interrogate, fast adaptive systems with frameworks designed for static devices are hardly governed by regulators, while LLMs actively propagate discredited race-corrected kidney function estimates into clinical consultations (15). As reported in (16–18) Fairness-blind CKD risk tools may silently delay specialist referral for Black and Hispanic patients. These limits have important clinical implications. This review offers a clinically focused synthesis of AI applications across nephrology domains while critically evaluating their methodological robustness and fragility, translational readiness, and future research significances.

We examined these interconnected domains in nephrology, which are: AKI prediction, CKD progression modelling, AI in nephropathology, an independent domain warranting keen synthesis, AI in kidney transplantation, AI in dialysis encompassing hemodialysis (HD) and peritoneal dialysis (PD), emerging AI methodologies (LLMs, RL, and federated learning), and the cross-cutting challenges such as interpretability, algorithmic fairness, and regulation (**Figure 1**).

**Figure 1:**
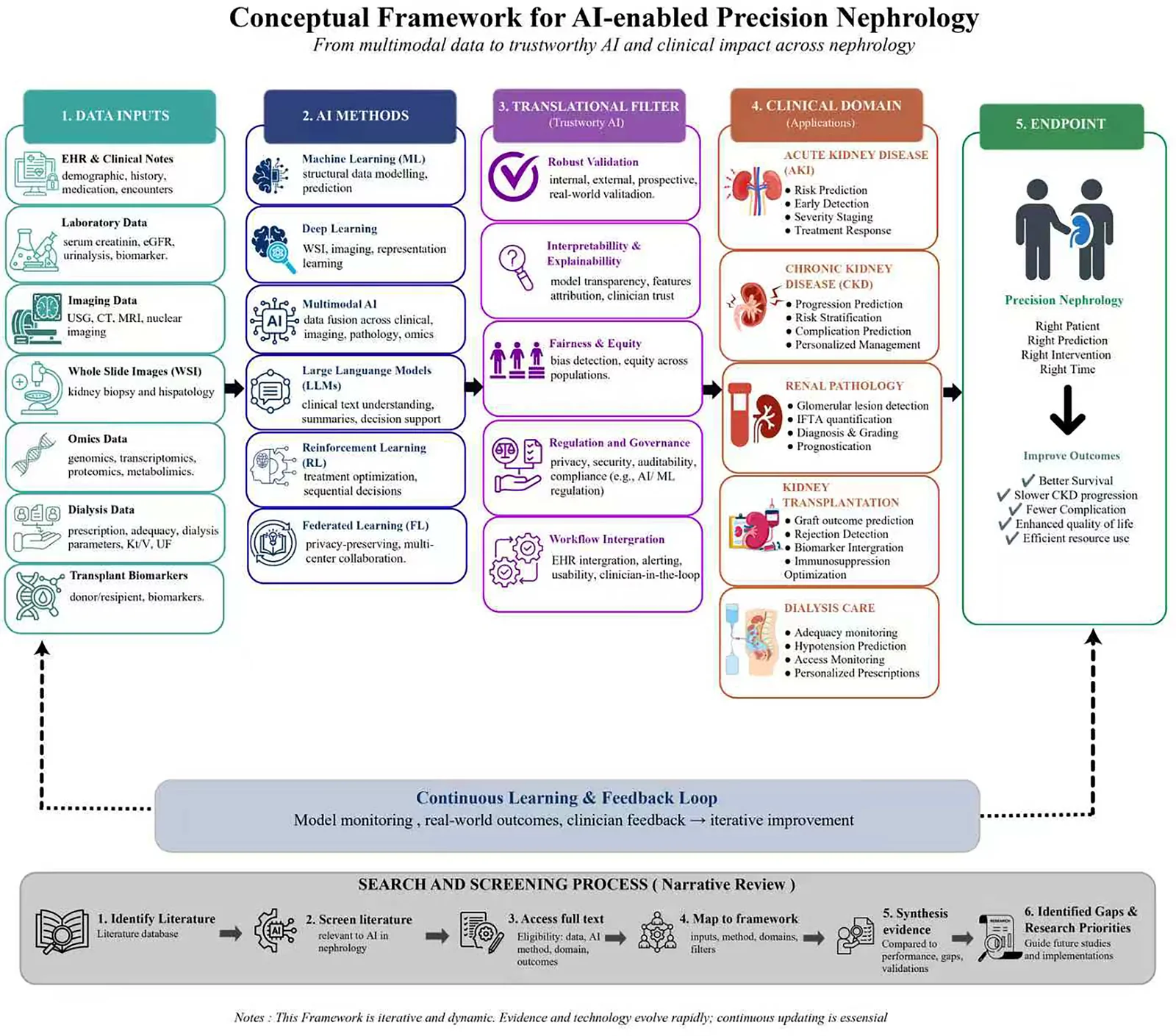
Conceptual framework for AI-enabled nephrology: from multimodal data to trustworthy AI and clinical impact across nephrology. The image illustrates how heterogeneous nephrology data sources are integrated through artificial intelligence methods. The framework ultimately links AI development to decision support, precision diagnosis, personalized treatment, workflow integration, and improved patient-centered nephrology outcomes.

## 2 Methodology

This review was conducted as a structured narrative review (19) guided by the Scale for the Assessment of Narrative Review Articles (SANRA) (20). A structured literature search was performed in PubMed on 24 November 2025. The search strategy combined terms related to artificial intelligence with terms covering kidney diseases, nephrology practice, and major nephrology applications. The artificial intelligence search block included “artificial intelligence,” AI, “machine learning,” “deep learning,” DL, “neural network,” “natural language processing,” NLP, “large language model*,” LLM, “generative AI,” “generative artificial intelligence,” “computer vision,” “reinforcement learning,” “federated learning,” and “transformer model*.” The nephrology search block included nephrology, “kidney disease*,” “renal disease*,” “acute kidney injury,” AKI, “chronic kidney disease,” CKD, “kidney failure,” “renal failure,” “end-stage kidney disease,” ESKD, “end-stage renal disease,” ESRD, glomerulonephritis, nephropathology, “kidney biopsy,” “renal biopsy,” “kidney transplant*,” “renal transplant*,” hemodialysis, haemodialysis, and “peritoneal dialysis.” The complete Boolean search strategy and database results are provided in the supplementary search documentation.

The eligibility criteria were defined *a priori* according to population/context, concept, clinical application, article type, publication period, language, and availability of full text. Studies were eligible if they addressed clinically relevant applications of artificial intelligence or related approaches in nephrology, including diagnosis, prediction, prognosis, nephropathology, kidney transplantation, dialysis, clinical decision support, or precision nephrology. Original research articles and relevant review articles published in English between 2020 and 2025 were eligible. Studies without clear relevance to nephrology or kidney health, studies using conventional statistical or computational methods without a meaningful artificial-intelligence component, purely technical studies without meaningful clinical or translational relevance, conference abstracts, protocols, letters, brief communications, publications lacking sufficient information for assessment, non-English publications, and duplicate records were excluded.

The PubMed search identified 9,179 records. Following title and abstract screening, 3,617 records were retained for full-text assessment. Title and abstract screening and subsequent full-text assessment were performed independently by MMI and CSA. Any disagreement between the two reviewers was resolved by discussion and, where consensus could not be reached, adjudicated by WA. The final eligible literature was thematically organized according to the major clinical domains of AI application in nephrology. Because this was a narrative review rather than a quantitative systematic review, studies most directly relevant to the predefined clinical domains and the review questions were selected for detailed narrative synthesis. The screening process and study selection were documented to support reproducibility (supplementary file).

Data were extracted from eligible studies according to their clinical domain and relevance to the review objectives. Extracted characteristics included study population and sample size, study design, clinical outcome or application, AI method, comparator where applicable, validation approach, reported performance measures, interpretability, fairness assessment, prospective evaluation, and evidence of clinical implementation or deployment when reported (**Table 1**). Where a characteristic was not reported in the source publication, it was recorded as “not reported” rather than inferred.

**Table 1:** Methodological characteristics of key included studies across AI in nephrology domains and AI technologies (2020–2025).

Study	Domain	Design/population	Outcome/horizon	AI method	Comparator	Validation	Performance	Calibration	Interpretability/fairness	Prospective evaluation	Deployment/regulatory	Key limitation
Tomašev et al. (2019)† (3)	AKI	Retrospective VA cohort; 703,782 patients	AKI up to 48 h before recognition	RNN	Conventional assessment/baseline models	Internal and subsequent external evaluation	AUROC 0.921	NR	NR	No prospective interventional evaluation	NR	Predominantly male cohort; generalizability concerns
Liu et al. (2024) (21)	CKD progression	Multicentre/multinational cohort	Kidney failure/death; longitudinal	KFRE + ML	KFRE	External population/recalibration	NR in current extraction	NR	NR	NR	NR	Incremental value beyond KFRE requires clearer demonstration
He et al. (2025) (6)	Early CKD	Multicentre cohort	Early-stage CKD	Interpretable ML/XGBoost	NR	Multicentre external validation	AUC 0.91	NR	Interpretability addressed; fairness NR	NR	NR	Clinical outcome benefit not demonstrated
Yoo et al. (2023) (9)	Nephropathology/transplant	Multicohort	Allograft histology classification	Deep learning + Banff molecular features	NR	Prospective multicohort	NR in current extraction	NR	NR	Prospective multicohort; not necessarily interventional	NR	Clinical utility/outcome benefit not established
Ye et al. (2024) (22)	Transplant rejection	Multicohort	Rejection subtype	Multiple-instance learning	NR	Independent test set	AUC 0.798 (rejection classification); AUC 0.936 (1-year graft-loss prognosis)	NR	NR	NR	NR	Independent testing; clinical utility not established
Divard et al. (2022) (23)	Transplant outcome	Multicohort	5-year graft failure	iBOX/multivariable DL	NR	International multicohort	NR in current extraction	NR	NR	NR	NR	Prospective clinical utility not established
Raynaud et al. (2021) (24)	Transplant outcome	International multicohort	Renal survival trajectory	Dynamic AI/longitudinal modelling	NR	International multicohort	AUC 0.857	NR	NR	NR	NR	Predictive performance does not establish improved graft outcomes
Lee et al. (2021) (25)	Haemodialysis	Retrospective; 261,647 HD sessions	Intradialytic hypotension within 1 h	Recurrent neural network (RNN)	NR	Internal train/validation/calibration/test	AUROC 0.94, 0.87 and 0.79	Reported as high	NR	NR	NR	Not reinforcement learning; no external/prospective validation
Yang et al. (2022) (26)	Haemodialysis	Retrospective data + simulation	Dry-weight optimisation	Dueling Double Deep Q-Network	Standard fluid-management protocols	Simulation + retrospective	NR in current extraction	NR	NR	No prospective RCT	NR	Sequential decision optimisation remains unvalidated prospectively
Miao et al. (2024) (27)	LLM/nephrology	Nephrology benchmark; 975 questions	Question accuracy	GPT-4/GPT-3.5	Nephrology examinee benchmark	Question-bank evaluation	GPT-4 74%; GPT-3.5 51%	NR	NR	No	NR	Knowledge benchmark; not clinical utility/patient benefit
Goh et al. (2024) (28)	General clinical LLM	RCT; 50 physicians	Diagnostic reasoning	GPT-4-assisted reasoning	Conventional resources	Prospective RCT	76%	NR	NR	Prospective RCT	NR	Not nephrology-specific; no significant improvement in physician reasoning
Pati et al. (2022) (29)‡	Federated learning	6,314 patients; 71 sites/6 continents	Tumour-boundary detection	Federated CNN	Centralised/public model	Cross-institutional federated evaluation	NR in current extraction	NR	Privacy-preserving architecture; fairness NR	NR	NR	Non-nephrology methodological evidence

*Pre-2020 foundational study, included via bidirectional citation tracking (Section 2).

^‡^A non-nephrology study included as a methodological reference for FL architecture.

(Section 9.3.4).

The evidence was synthesized thematically across acute kidney injury, chronic kidney disease, nephropathology, kidney transplantation, haemodialysis, peritoneal dialysis, and emerging AI approaches including large language models, reinforcement learning, and federated learning. Particular attention was given to the distinction between model development, internal validation, external validation, prospective clinical evaluation, clinical utility, and actual implementation or deployment. Predictive performance measures were therefore not interpreted in isolation as evidence of clinical utility or patient benefit. The evidence-maturity and translational-readiness framework described in Section 10 was used as a qualitative synthesis tool to describe the relative maturity and readiness of evidence across domains; it was not treated as a validated numerical scoring instrument or as a threshold-based measure of clinical deployment.

Two foundational pre-2020 studies were additionally considered because of their methodological importance to the development of AI applications in nephrology and were identified through citation chaining. These studies were treated as foundational evidence outside the predefined 2020–2025 database-search period and were distinguished from studies identified through the primary database search. Regulatory and policy documents were considered separately as supplementary sources where necessary to contextualize issues of AI governance, reporting, and clinical implementation (30–32).

## 3 AI in acute kidney injury: prediction to clinical decision support

### 3.1 Decision support

In various aspects, AKI is the paradigmatic AI target in nephrology. The nephrology field took a forward decisive step when (3) demonstrated that a recurrent neural network (RNN) trained on 703,782 Department of Veterans Affairs patients (VA) can predict 55.8% of inpatient AKI episodes up to 48 hours before clinical recognition, with an AUROC of 92.1% for any AKI stage. That single result proved a performance benchmark and set the scrutiny standard for studies that followed.

Successive multinational studies have proven generalizable accuracy while exposing its limits (4). delivered the largest external validation outside the VA ecosystem 16,760 ICU patients across 145 units in the United States, the Netherlands, and Italy achieving an AUROC of 0.884 for Kidney Disease: Improving Global Outcomes (KDIGO) stage 2/3 AKI with greater than 14 hours of lead time. The same group extended this to persistent AKI stage 3, using an interpretable tree regressor which is validated in two independent multicenter cohorts (33), although generalizability is not guaranteed. The (3) cohort was 93.6% male, and the models trained on it underperformed in female populations, while (34, 35) documented reproducibility failures across externally validated AKI models, confirming that impressive development cohort AUROCs must not be extrapolated uncritically. A prospective evidence gap remains evident (36). represents the field’s closest approach to closing it, a prospective multicenter pediatric ICU cohort study translating a Shapley Additive exPlanations (SHAP) that interpreted a random forest model into a real-time clinical web application with demonstrated feasibility and clinical usability confirmed, but with reduced dialysis dependence, and improved mortality never measured, is the field’s central unfinished task.

Progress on implementation barriers continues (37), reported a point-of-care ML model reaching an AUC of 0.85, a recall of 0.63, and a precision score of 0.54, using only features available at emergency presentation, sidestepping the baseline creatinine problem that plagues admission-stage deployment, while (38) identified reinforcement learning for adaptive dialysis optimization as an emerging technique in critical care nephrology, extending AI application from early AKI detection into adaptive therapeutic management. AKI subphenotyping deserves recognition as a distinct and clinically consequential advance, not merely a methodological curiosity. In (39) latent class analysis was used to identify two molecular AKI subphenotypes with divergent responses to vasopressin therapy, demonstrating that phenotypic enrichment can convert a null trial result into a genuine treatment signal (40). formalized the conceptual distinction between prognostic and predictive subphenotypes, while (41) extended biomarker-driven subphenotyping toward targeted intervention pathways and (42) combined machine learning with target trial emulation to identify vasopressin-responsive versus norepinephrine-responsive clusters using a rigorous causal-inference framework, representing a methodologically rigorous approach to AKI subphenotyping.

Early AKI prediction was enhanced by incorporating urinary kidney injury biomarkers into an interpretable eXtreme Gradient Boosting (XGBoost) model. Their model (AKIML^pred^), integrating nine biomarkers including neutrophil gelatinase associated lipocalin (NGAL), kidney injury molecule 1 (KIM-1), insulin-like growth factor-binding protein 7 (IGFBP7), interleukin 18 (IL-18), liver fatty acid binding protein (L-FABP), cystatin C, c-terminal fragment 22 of agrin (CAF22), and N-terminal pro-brain natriuretic peptide (NT-proBNP), achieved an AUC of 0.902 in an external test cohort and an accuracy of 0.846 and outperformed models based solely on routine clinical variables. The model predictions were interpreted using SHAP algorithm analysis, enhancing clinical transparency (43).

## 4 AI in chronic kidney disease

AI-based CKD prediction has increasingly incorporated longitudinal and multimodal data (44–46), but its clinical value should be judged against established risk-prediction tools. Liu et al. evaluated and recalibrated the Kidney Failure Risk Equation (KFRE) in a multinational, population-based cohort, providing an important benchmark for assessing newer prediction models (21). Machine-learning approaches have subsequently explored additional predictors and more complex modelling strategies (47–49), including personalized prediction of CKD progression (50), TabNet and LSTM-based CKD detection (51), and multimodal fusion models for CKD progression prediction (52–55). These approaches may capture nonlinear relationships and longitudinal patterns that are not fully represented by conventional risk equations. However, higher discrimination alone does not establish clinically meaningful incremental value over KFRE. Evidence of improved calibration, reclassification, decision-analytic performance, or patient outcomes would be needed to justify replacing or routinely supplementing established tools. Current evidence therefore supports AI as a promising complement to conventional CKD risk prediction rather than establishing its superiority for clinical decision-making (21, 50, 51, 56).

## 5 AI in nephropathology

AI applications in nephropathology have focused on automated analysis of kidney biopsy images, lesion classification, fibrosis quantification, and transplant pathology. A multicentre proof-of-concept study by Kers et al. applied deep learning to kidney transplant pathology and demonstrated classification of biopsies into normal, rejection, and other categories, with reported AUC values of 0.80–0.87 (7). Other work has explored automated quantification of IgA nephropathy histological features using deep sequential neural networks (57) and deep-learning quantification of interstitial fibrosis in digitized kidney biopsies (58). AI-based approaches have also been investigated for ultrastructural assessment of glomerular morphology, although these applications remain comparatively early (59, 60). More broadly, foundation-model approaches may reduce dependence on extensive manual annotation, but their nephrology-specific external validation remains limited (61). Thus, although nephropathology represents a promising area for AI-assisted decision support (62), current evidence does not establish that these systems can replace specialist pathological assessment or improve patient outcomes (**Figure 2**).

**Figure 2:**
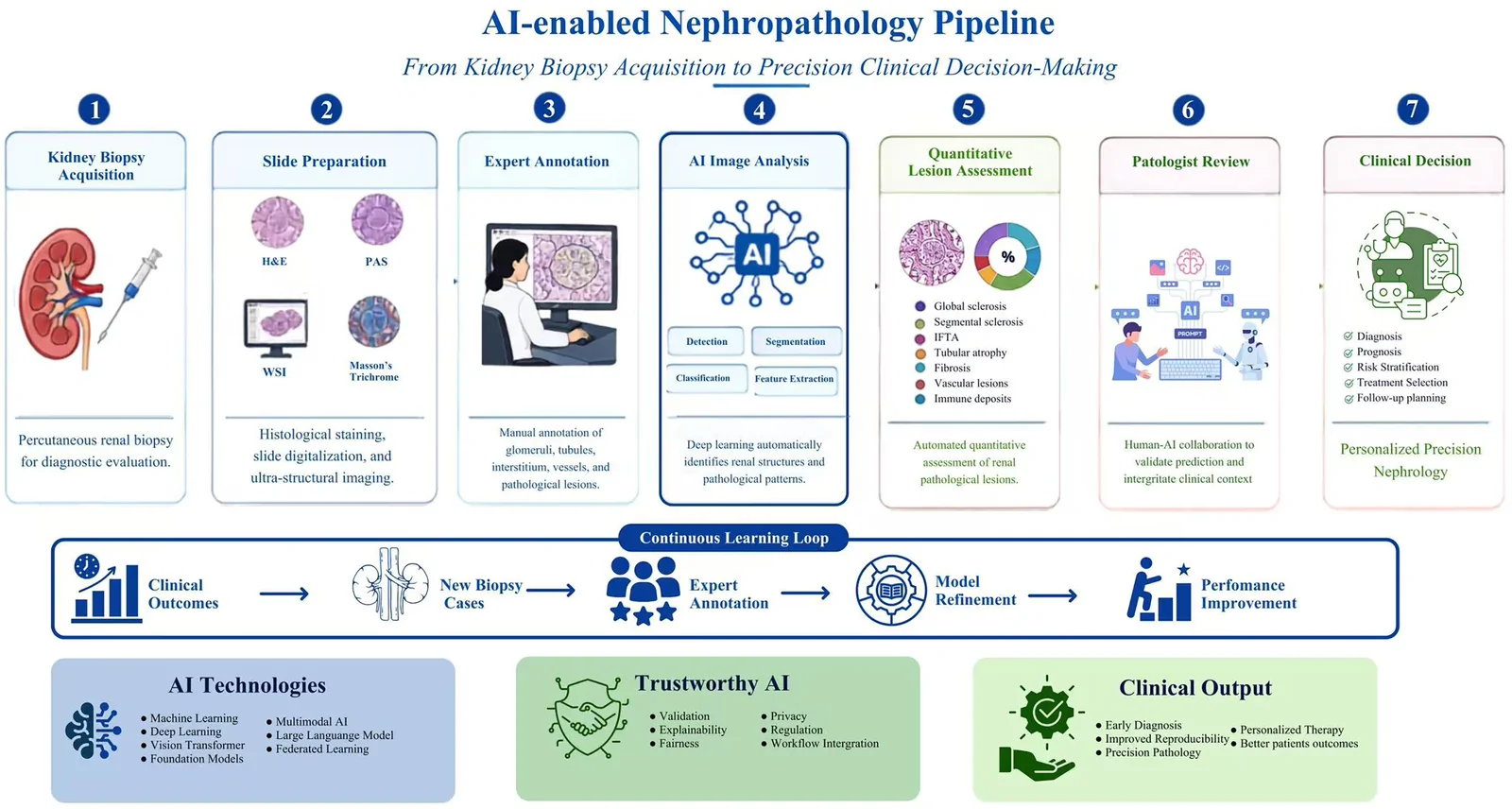
AI-enabled nephropathology pipeline. This image illustrates an overview of AI application domains, methods, and pipeline usage in nephropathology, from kidney biopsy acquisition to clinical decisions leading to personal precision nephrology.

## 6 AI in kidney transplantation

AI applications in kidney transplantation include graft-failure prediction, rejection classification, dynamic risk estimation, and digital pathology (63). The iBox system integrated clinical and transplant-related information to predict long-term kidney allograft failure and was compared with predictions made by transplant physicians (23). Deep-learning approaches have also been evaluated for kidney allograft histology, including automated classification systems based on morphological and molecular Banff features (9) and independent evaluation of whole-slide-image models for rejection classification (22). Dynamic AI-based prediction of renal survival has additionally been evaluated in an international multicohort observational study (24). These studies indicate substantial progress in external and multicohort evaluation, but predictive or diagnostic performance should not be interpreted as evidence that AI improves transplant decision-making or graft outcomes. Prospective interventional evaluation remains necessary before claims of routine clinical deployment can be justified (9, 22–24).

## 7 Haemodialysis

AI applications in haemodialysis include prediction of intradialytic hypotension and optimisation of dialysis-related decisions. A recurrent neural network evaluated in 261,647 haemodialysis sessions achieved an AUROC of 0.94 for one definition of intradialytic hypotension, with additional AUROCs of 0.87 and 0.79 for alternative definitions (25). This represents strong retrospective predictive performance, but it does not establish prospective clinical utility or improved patient outcomes. In contrast, reinforcement learning has been applied to dry-weight assessment using a dueling double deep Q-network (Duel-DDQN), framing dry-weight optimisation as a sequential decision-making problem (26). The latter approach should therefore be distinguished from the RNN-based prediction model rather than classified as the same AI methodology. Other applications include AI-assisted anaemia management and CKD-mineral and bone disorder treatment optimisation (64, 65), while machine-learning approaches have also been explored for dialysis adequacy prediction (66). Overall, the evidence remains predominantly retrospective, simulated, or otherwise limited in prospective evaluation, and high predictive performance should not be equated with clinical deployment.

## 8 Peritoneal dialysis

AI research in peritoneal dialysis remains substantially less developed than that in haemodialysis. Prediction models for peritoneal dialysis-associated peritonitis have reported individual AUCs ranging from 0.659 to 0.997, although a systematic review and meta-analysis identified substantial methodological limitations and a high risk of bias across the available models (67). Machine learning has also been applied to prediction of technique failure following peritoneal dialysis-associated peritonitis. A random-forest model reported an AUC of 0.916, with accuracy of 93.70%, sensitivity of 96.67%, and specificity of 86.49% in a retrospective cohort of 508 episodes (68). These results demonstrate promising discrimination but do not establish external validity or clinical benefit. The limited number of multicentre and prospective studies remains a major barrier to translation (**Figure 3**).

**Figure 3:**
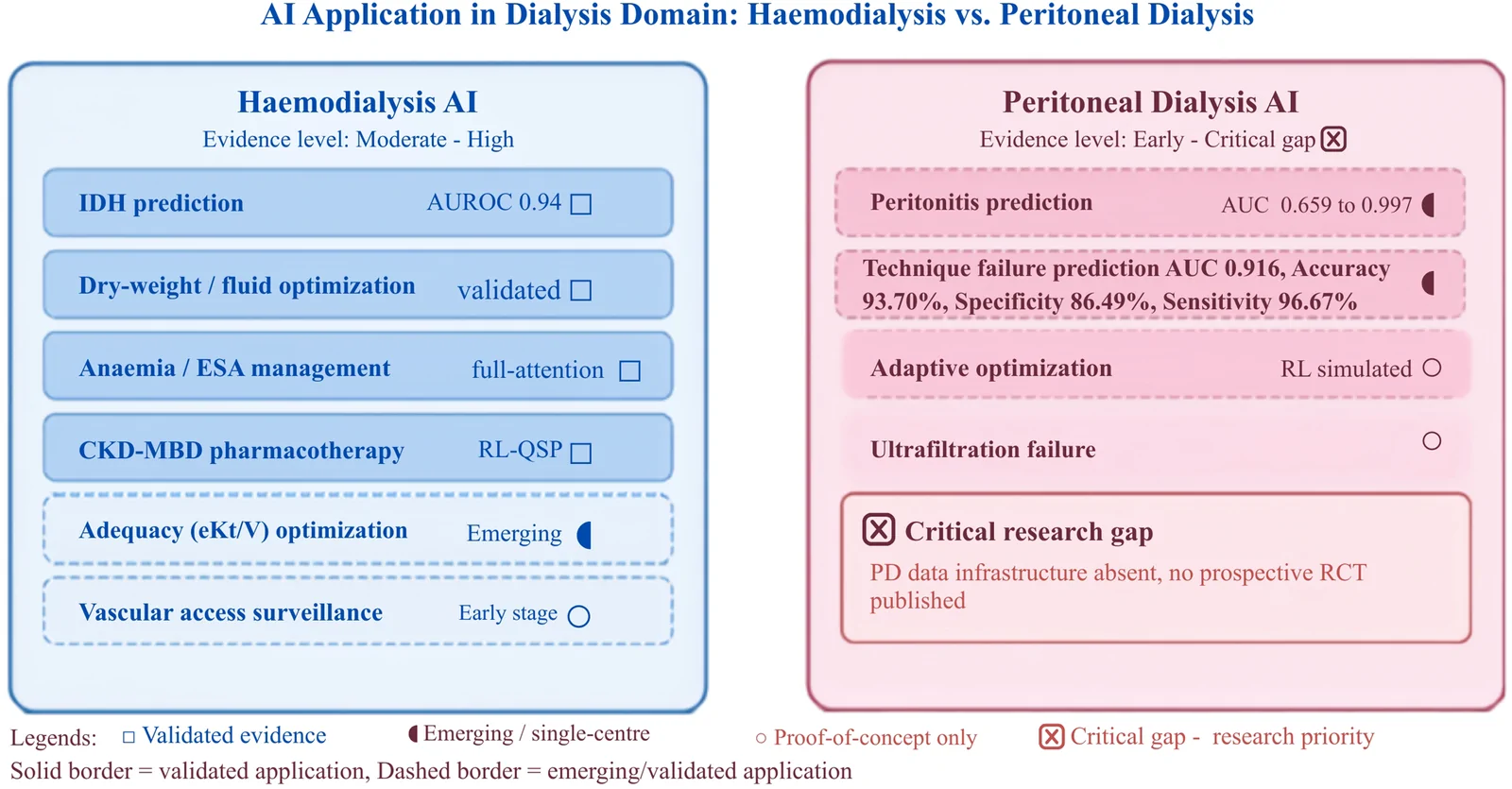
AI application and current evidence maturity for both hemodialysis and peritoneal dialysis modalities. The image illustrates evidence maturity between hemodialysis and peritoneal in kidney dialysis, analyzing validated evidence, emerging evidence, proof of concept, and critical gaps.

## 9 Evidence synthesis, maturity, and future research directions

### 9.1 Qualitative evidence-maturity and translational-readiness framework

The evidence identified across AI applications in nephrology varies considerably in methodological maturity and translational relevance. To support a balanced synthesis, we considered evidence maturity and translational readiness as related but distinct concepts. Evidence maturity reflects the strength and consistency of the available evidence, including study design, population size, validation strategy, and reproducibility across settings. Translational readiness considers the extent to which an AI application has progressed from model development and retrospective validation toward prospective clinical evaluation, demonstrated clinical utility, and integration into clinical workflows.

The framework is a qualitative synthesis tool for describing evidence maturity and translational readiness across nephrology AI applications. It is not a validated numerical scoring instrument and does not assign a numerical threshold for clinical deployment. High discrimination or successful external validation alone was therefore not considered sufficient evidence for clinical deployment. Particular attention was given to external and prospective evaluation, calibration and interpretability, fairness considerations, clinical utility, and evidence of impact on clinical decision-making or patient outcomes. The assessment dimensions and their evaluation guidance are provided in **Table 2** (**Figure 4**).

**Table 2:** Qualitative evidence-maturity and translational-readiness framework by nephrology domain*.

Clinical/technological domain	Evidence maturity	External validation	Calibration/interpretability	Prospective clinical evaluation	Clinical utility/patient benefit	Translational readiness	Principal limitation
AKI prediction	Moderate–high	Present for selected models	Inconsistently reported	Limited	Not established by prospective intervention	Emerging	Strong retrospective discrimination in selected cohorts, but limited prospective evidence and uncertainty regarding impact on clinical decisions and outcomes
CKD progression/risk prediction	Moderate	Present in selected studies	Variable; interpretability increasingly addressed	Limited	Incremental benefit beyond established tools such as KFRE remains insufficiently established	Emerging	High predictive performance does not necessarily demonstrate clinically meaningful improvement over validated conventional risk tools
Nephropathology	Moderate–high	Present in multicentre/multicohort studies	Variable	Limited	Not established through prospective intervention	Emerging	Dataset and center dependence, annotation requirements, and limited evidence that AI-assisted interpretation improves patient outcomes
Kidney transplantation	Moderate–high	Present for selected models	Variable	Limited	Not established	Emerging	Heterogeneity in populations, endpoints and validation strategies; prospective clinical utility remains limited
Haemodialysis	Moderate	Limited	Variable	Limited	Not established	Emerging	Most evidence remains retrospective or simulation-based; decision-support models require prospective evaluation
Peritoneal dialysis	Early–moderate	Limited	Inconsistently reported	Limited	Not established	Early/emerging	Relatively smaller evidence base and limited external/prospective validation
Large language models	Early–moderate	Benchmark and task-specific evaluation	Limited clinical calibration evidence	Limited nephrology-specific prospective evidence	Not established for routine nephrology care	Early/emerging	Benchmark performance does not establish reliability, clinical utility, safety or patient benefit
Reinforcement learning	Early–moderate	Limited	Variable	Very limited	Not established	Early	Predominantly retrospective/simulation evidence; prospective safety and effectiveness remain uncertain
Federated learning	Early	Cross-institutional methodological evidence	Model-dependent	Limited nephrology-specific evidence	Not established	Early	Evidence is largely methodological or from non-nephrology applications; nephrology-specific clinical validation remains limited

*Assessment guidance for **Table 2**: Evidence maturity reflects the overall robustness and consistency of the evidence, considering study design, population, validation, and reproducibility. External validation refers to testing in an independent population, institution, or dataset outside the development cohort. Calibration/interpretability refers to agreement between predicted and observed risk and the transparency or understandability of model outputs. Prospective clinical evaluation refers to prospective assessment in real-world clinical settings, preferably with evaluation of clinical decisions or outcomes. Clinical utility/patient benefit refers to demonstrated improvement in clinical decision-making, workflow, safety, or patient-centered outcomes attributable to AI use. Translational readiness is a qualitative judgement of progress toward safe and feasible real-world integration, informed by validation, utility, implementation, and governance. Principal limitation records the main factor constraining confidence or translation.

**Figure 4:**
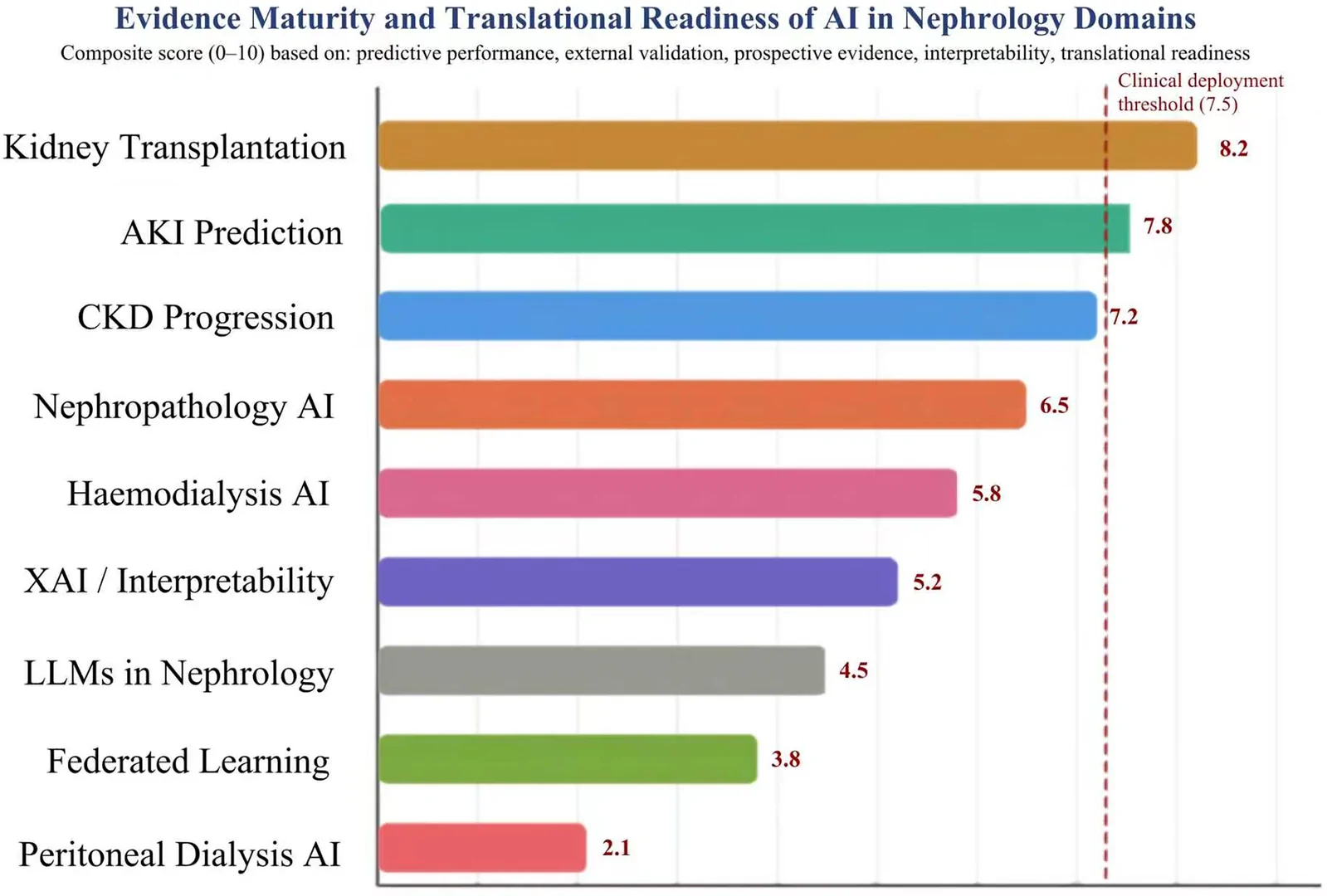
Qualitative evidence maturity and translational readiness of AI in nephrology domains. This image summarizes the qualitative evidence-maturity and translational-readiness assessment across major AI domains; the categories are descriptive and are not numerical scores or validated clinical-deployment thresholds.

### 9.2 Areas of convergence and evidence-based consensus

Several consistent findings emerge from the available literature. AI models demonstrate promising performance across multiple nephrology applications, particularly AKI and CKD prediction, although performance and generalizability vary substantially between populations and validation settings. Systematic evidence in CKD similarly demonstrates substantial heterogeneity in algorithms, datasets, outcomes, and methodological quality, limiting direct comparison between models (69).

Multimodal approaches may provide additional opportunities by integrating clinical, imaging, laboratory, and longitudinal information. However, their clinical value depends on validation beyond the development population and demonstration that the additional information produces meaningful improvement over existing approaches (70).

Interpretability is another recurring consideration. Although explainable AI may facilitate clinician understanding and trust, explanations do not uniformly increase trust and may have different effects depending on their quality and context (71). Across these domains, the available evidence therefore supports considerable technical promise but remains insufficient to equate model performance with established clinical benefit.

### 9.3 Original perspectives and future research directions

We propose six future research priorities that address significant gaps in the field, based on the current evidence (**Table 3**).

**Table 3:** Proposed priority future research agenda for AI in nephrology.

Priority	Research question	Specific recommendation	Timeline*
1. Prospective Trials	Does AI prediction change nephrology outcomes?	Establish a precompetitive trial framework with standardized AKI/CKD/dialysis endpoints and prioritize transplant pathology AI RCT	2–3 years
2. Nephropathology AI	Can AI-assisted biopsy improve diagnostic and treatment decisions?	Build an international multicenter annotated WSI registry and trial AI-assisted vs conventional biopsy interpretation for lupus nephritis and IgA nephropathy.	3–5 years
3. Peritoneal Dialysis AI	Can AI optimize PD prescription and predict peritonitis and technique failure?	Develop standardized PD electronic data capture and multicenter retrospective validation studies. Develop an RL-based adaptive PD prescription framework.	2–4 years
4. Federated Governance	Can governance infrastructure enable FL across nephrology institutions?	Adapt the Kidney Precision Medicine Project governance model for FL and establish legal and attribution frameworks across the EU, the United States (US), and low-and middle-income countries’ (LMIC) regulatory regimes.	3–5 years
5. Human-AI Integration	What AI tool designs and interface features improve clinical decisions?	Randomized simulated trial methodology, human factors engineering program, and implementation of science studies across AKI, CKD, and dialysis AI domains	2–4 years
6. Global Equity in AI	Can nephrology AI achieve equitable performance across diverse global populations?	Mandate demographic diversity reporting in all AI nephrology publications and establish LMIC specific model development and validation consortia.	Ongoing

*The estimated timelines reflect the authors proposed expert opinion and are not entirely derived evidence.

#### 9.3.1 The prospective trial imperative: from accuracy to outcome benefit

The next stage of nephrology AI research should move beyond demonstrating predictive accuracy toward determining whether AI-assisted care improves clinical decisions and patient outcomes. This is particularly important because retrospective validation cannot establish the effect of using an AI system in routine clinical practice. AKI, CKD, and dialysis therefore require prospective studies that evaluate not only discrimination but also changes in clinical management, safety, and patient-centered outcomes.

#### 9.3.2 Nephropathology AI: from research tool to clinical decision support

Nephropathology AI should progress from proof-of-concept image analysis toward prospective evaluation of its effect on diagnostic consistency and clinical decision-making. Multicentre datasets incorporating variation in institutions, scanners, staining procedures, and patient populations will be important for assessing generalizability. Future studies should also determine whether AI assistance changes diagnostic classification, treatment decisions, or other clinically relevant outcomes.

#### 9.3.3 Peritoneal dialysis AI: a research priority proportionate to clinical need

The relatively limited evidence base for AI in peritoneal dialysis warrants greater development of multicentre longitudinal datasets and standardized outcome definitions (72–74). Future research should evaluate prediction of peritonitis, technique failure, and other clinically relevant outcomes, followed by prospective assessment of whether AI-supported decision-making improves patient outcomes. High retrospective predictive performance should not, by itself, be interpreted as evidence of clinical utility.

#### 9.3.4 Federated nephrology AI: governance before technology

Federated learning provides a potential framework for collaborative AI development without requiring centralization of patient-level data (75). However, successful technical implementation does not by itself establish clinical readiness. Future nephrology applications should address data governance, privacy, interoperability, institutional responsibilities, auditability, and performance across participating centres. Existing federated-learning evidence provides important methodological support, although much of the literature is not nephrology-specific (29, 76).

#### 9.3.5 The comprehensiveness principle

AKI and CKD remain the dominant focus of nephrology AI research, while comparatively less attention has been directed toward nephropathology, dialysis modalities, disease heterogeneity, and treatment optimisation. Future research should broaden the evidence base across these areas while ensuring adequate representation of diverse populations and healthcare settings. Such expansion is necessary for AI nephrology to reflect the full spectrum of clinical practice rather than a limited number of well-studied applications.

#### 9.3.6 Human AI integration science: the missing research agenda

Understanding how clinicians interact with AI outputs under real-world cognitive and temporal constraints remains an important research gap. In a randomized clinical trial, physician–LLM teams did not demonstrate superior diagnostic reasoning compared with physicians using conventional resources, highlighting that access to an AI system does not automatically produce better clinical reasoning (28). Similarly, systematic evidence indicates that explainable AI can either increase or decrease clinician trust depending on how explanations are presented and interpreted (71).

These findings support a dedicated research agenda focused on human–AI interaction, workflow integration, clinician oversight, and human-factors engineering. Future nephrology studies should therefore evaluate not only whether an AI system is accurate, but also how its outputs are incorporated into clinical reasoning and whether that interaction improves care.

## 10 Limitations of this review and the current literature

Five important limitations characterize the current evidence base. First, many AI models are developed retrospectively and evaluated using internal validation, limiting assessment of real-world performance and clinical utility. Second, generalizability remains a concern because many datasets do not adequately represent populations from different geographic, demographic, and healthcare settings. Third, reporting of calibration, interpretability, and other clinically relevant performance characteristics remains inconsistent (77).

Fourth, implementation may be constrained by infrastructure, computational requirements, data availability, and differences in healthcare resources across settings. Fifth, evidence regarding cost-effectiveness and health-system impact remains limited.

This review also has limitations related to the breadth and rapid evolution of the field. Evidence for some applications, particularly peritoneal dialysis AI, remains relatively limited compared with AKI and CKD. In addition, the rapid development of large language models and related technologies means that new evidence may emerge after the literature-search period. These limitations reinforce the need for continued prospective and multicentre evaluation rather than diminishing the potential relevance of AI to nephrology. The evidence-maturity and translational-readiness framework also has an inherent degree of subjectivity because it synthesizes heterogeneous study designs, outcomes, and clinical contexts. It has not been formally validated as a measurement instrument, and its domain ratings should therefore be interpreted as a structured qualitative synthesis rather than objective scores.

## 11 Conclusion

AI is increasingly being applied across nephrology, including AKI prediction, CKD risk assessment, nephropathology, transplantation, haemodialysis, peritoneal dialysis, large language models, reinforcement learning, and federated learning. The available evidence demonstrates substantial technical progress, but evidence maturity and translational readiness vary considerably across applications.

The key challenge is now to establish whether promising AI performance translates into reliable clinical decision-making and meaningful patient benefit. External validation, prospective evaluation, appropriate assessment of calibration and fairness, interpretability, regulatory oversight, and effective human-AI integration will therefore be essential for safe translation. AI should complement rather than replace clinical expertise, with its ultimate value determined by demonstrated benefit within real-world nephrology practice.

Without AI, precision nephrology cannot match biological, clinical, and contextual patient profiles to optimal intervention. But AI is clearly insufficient on its own, the nephrology research community will require sustained and coordinated engagement to achieve this goal.

## Glossary

AI: artificial intelligence
AKI: acute kidney injury
ANCA: anti-neutrophil cytoplasmic antibody
AUROC: area under the receiver operating characteristic
AUC: area under the curve
AVF: Arteriovenous Fistula
CT: Computed Tomography
CKD: chronic kidney disease
CI: Confidence Interval
CKD-EPI: Chronic Kidney Disease Epidemiology Collaboration
CAF22: c-terminal fragment 22 of agrin
CKD-MBD: chronic kidney disease–Mineral and Bone Disorder
CNN: Convolutional neural networks
DL: deep learning
Duel-DDQN: Dueling double Deep Q-Network
eGFR: estimated Glomerular Filtration Rate
ESRD: end stage renal disease
EU: European Union
EMA: European Medicines Agency
ESA: Erythropoiesis-Stimulating Agent
EHR: electronic health record
FDA: Food and Drug Administration
FL: Federated Learning
PD: peritoneal dialysis
GBM: glomerular basement membrane
GRU: Gated Recurrent Unit
H&E: Hematoxylin and Eosin
HD: hemodialysis
ICU: Intensive Care Unit
IGFBP7: insulin-like growth factor-binding protein 7
IL-18: interleukin 18
IEEE: Institute of Electrical and Electronics Engineers
IDH: intradialytic hypotension
IoMT-AI: Internet of Medical Things - Artificial Intelligence
IFTA: Interstitial Fibrosis and Tubular Atrophy
KIM-1: kidney injury molecule 1
KDIGO: Kidney Disease: Improving Global Outcomes
KFRE: Kidney Failure Risk Equation
RNN: Recurrent Neural Network
LSTM: Long Short-Term Memory
LLM(s): large language models
L-FABP: liver fatty acid binding protein
LMIC: Low and Middle Income Countries
RL: Reinforcement learning
ML: machine learning
SHAP: SHapley Additive exPlanations
US: United States
WSI: whole slide images
RCT: Randomised Controlled Trial
VA: Department of Veterans Affairs
PICU: Pediatrics Intensive Care Unit
TEM: Transmission Electron Microscopy
NKF-ASN: National Kidney Foundation-American Society of Nephrology
TRIPOD-AI: Transparent Reporting of a multivariable prediction model for Individual Prognosis Or Diagnosis–AI extension
XAI: Explainable Artificial Intelligence
NGAL: neutrophil gelatinase-associated lipocalin
NT-proBNP: N-terminal pro-brain natriuretic peptide
RRT: Renal Replacement Therapy
PASM: Periodic Acid–Silver Methenamine
PAS: Periodic Acid-Schiff
FSGS: Focal Segmental Glomerulosclerosis
MEST-C: mesangial (M) and endocapillary (E) hypercellularity, segmental sclerosis (S) and interstitial fibrosis/tubular atrophy (T), crescents (C)
PDAP: peritoneal dialysis-associated peritonitis
NICE: National Institute for Health and Care Excellence
TRIPOD-LLM: TRIPOD extension for Large Language Model
NMPA: National Medical Products Administration
κ: kappa
SaMD: software as a medical device
MD: Medical Doctor
